# Human-Designed Filters May Outperform Machine-Learned Filters

**Published:** 2022

**Authors:** Gengsheng L Zeng

**Affiliations:** 1 Utah Valley University, Orem, Utah, 84058, USA; 2 University of Utah, Salt Lake City, Utah, 84108, USA

**Keywords:** Data science, Denoising, Image processing, Machine learning, Nonlinearity, Signal processing

## Abstract

Machine-learned image processing systems in medical imaging have shown better results than those obtained by traditional human-designed techniques. The success of machine learning techniques inspires humans to design better systems. The convolutional neural network (CNN) has a multi-channel architecture, which the conventional filters do not have. This paper proposes that by borrowing the multi-channel architecture, the human-designed denoising filter can have better performance than the machined-learned version. We illustrate the feasibility of this idea with a toy example in a sinogram denoising task in the area of tomography.

## Introduction

Machine learning is believed to have the potential to outperform the conventional technologies [1]. Without doubt, machine learning is one of the most exciting technologies of today. In the medical imaging industry, the FDA has approved machine learning image reconstruction techniques for clinical usage, because the machine learning based techniques can provide images with less noise and higher spatial resolution [2].

We believe that the superius performance of machine learning induced methods is due to its nonlinearity. On the other hand, classical methods are mostly based on linear models, such as the well-known filtered back-projection (FBP) algorithm. If the objective function is quadratic, the iterative gradient descent algorithm is a linear algorithm. When some nonlinearity is introduced to medical image reconstruction, the reconstructed images have some desirable properties that linear methods do not have. For example, the total variation (TV) constrained image reconstruction is a nonlinear method, which is able to remove noise and maintain sharp edges [3]. Some imaging problems can be modeled as compressed sensing problems, and their solutions rely on nonlinear L_1_ optimization [4].

The power of deep learning relies on the nonlinear activation function in every layer of the neural network. Without the nonlinear functions, the entire neural network will degenerate to a one-layer linear network. This current paper is inspired by the architecture of neural networks. We present a toy example, in which the human designed filter uses the architecture of a neural network.

## Methods and Results

The convolutional neural network (CNN) has wide applications in image processing [5]. The U-net version of it is popular in the segmentation applications [6]. CNN has a unique feature of using multiple channels. The development of a neural network today is still empirical. The network architecture and super parameter selection are tuned by trial and error.

**Figure 1:**
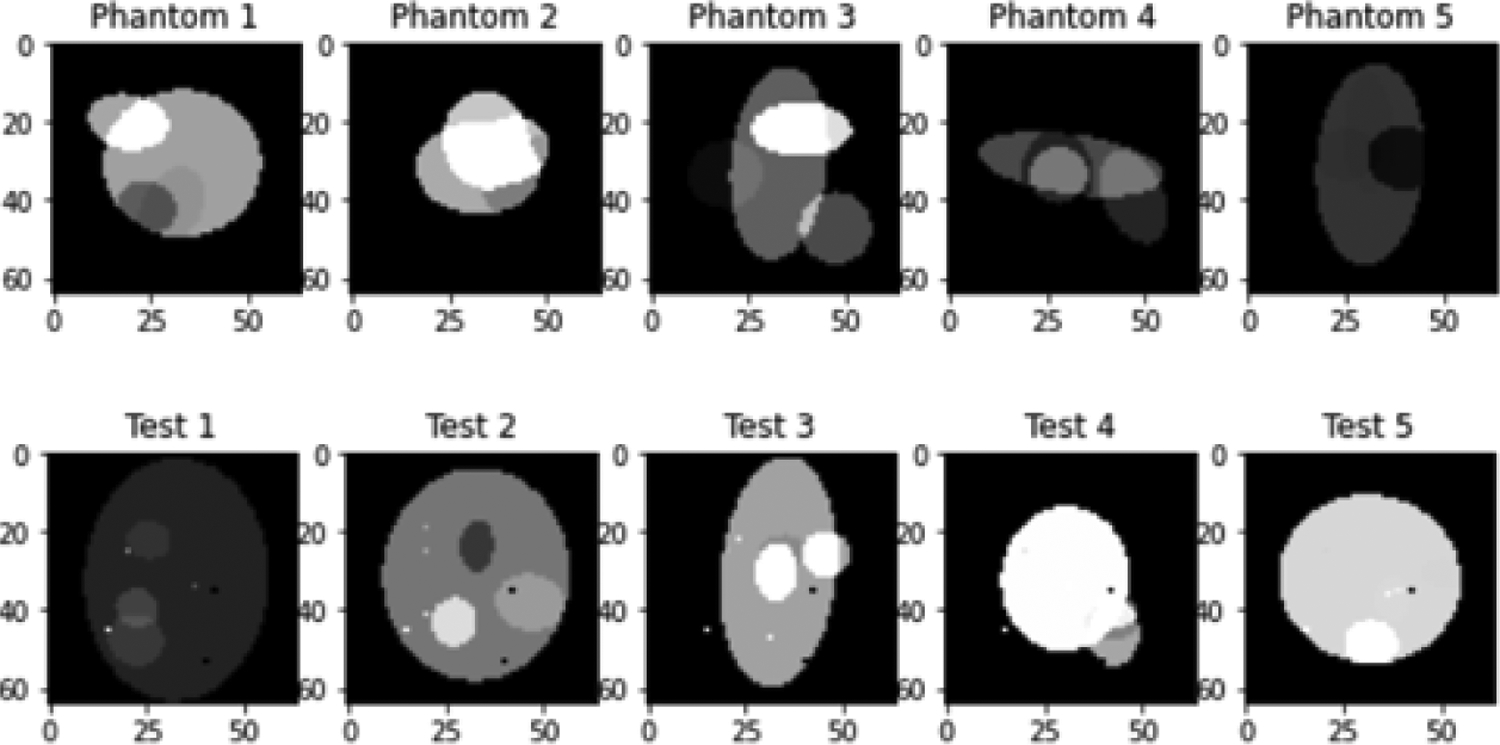
Some random phantoms. Top: For training. Bottom: For testing.

The toy example in this paper is sinogram denoising using computer simulations. The detector had 64 bins. The number of views over 360° was 360. The two-dimensional (2D) image reconstruction algorithm was FBP. Additive zero-mean Gaussian noise was added to the sinograms. The number of random sinograms was 1000 used in the CNN training, and the number of random unseen testing phantoms was 10. The testing phantoms contained some small dots that were not generated in the training data. The convolution kernels in the CNN were all 3 × 3. The nonlinear activation function in the CNN was the rectified linear unit (ReLU) function. The number of training epochs was 100. Some typical random phantoms are shown in Fig. 1, which contains ellipses with various sizes, locations, and intensity values. The testing errors are calculated and reported here in terms of the mean squared error (MSE) in the sinogram domain, with respect to the noiseless true sinograms. From our studies, the bias term in the neurons is not effective for the denoising task and is thus discarded in all our neural networks in this paper (Figure 1).

### One-Channel Multilayer CNN

Table 1 shows some typical testing study MSE values of the one-channel CNN experiments. As the number of layers increases, the MSE values do not have a decreasing trend (Table 1).

As a comparison, Table 2 lists the results of the one-layer, one-channel network with various convolution kernel sizes. This method is equivalent to the conventional linear filtering (Table 2).

### Multi-Channel Two-Layer CNN Network

Table 3 shows the results of two-layer CNN results. The first layer has multiple channels; the second layer has one channel. It is observed from Tables 1 and 3 that using a multi-channel shallow network is better than using a one-channel multi-layer network (Table 3).

### A Human-Designed Denoising Filter

**Figure 2:**
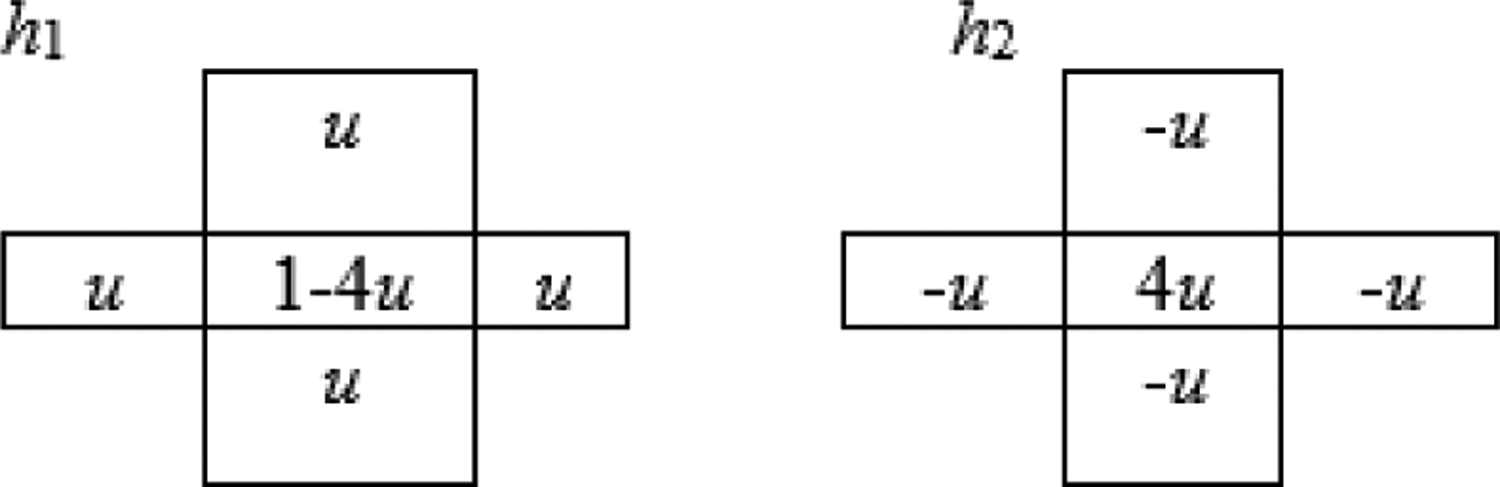
Convolution kernels for our human-designed filter.

The traditional human-designed filters have one layer and one channel. Inspired by the multi-channel CNN, we propose a two-channel filter, whose input image is f and output image are g. The input-output relationship is expressed as

$$\text{g}=\left[\sigma \left(\text{f}*{\text{h}}_{1}\right)-\sigma \left(\text{f}*{\text{h}}_{2}\right)\right]*{\text{h}}_{1},$$

where h_1_ is a 2D low-pass filter convolution kernel, h_2_ is a 2D high-pass filter convolution kernel, and σ is the ReLU function. The two terms inside the square brackets in (1) are considered as the first layer neurons, and the convolution with h_1_ at the end is the second layer. In our design, there is only one design parameter, u = 0.1, as shown in Fig. 2 (Figure 2).

**Figure 3:**
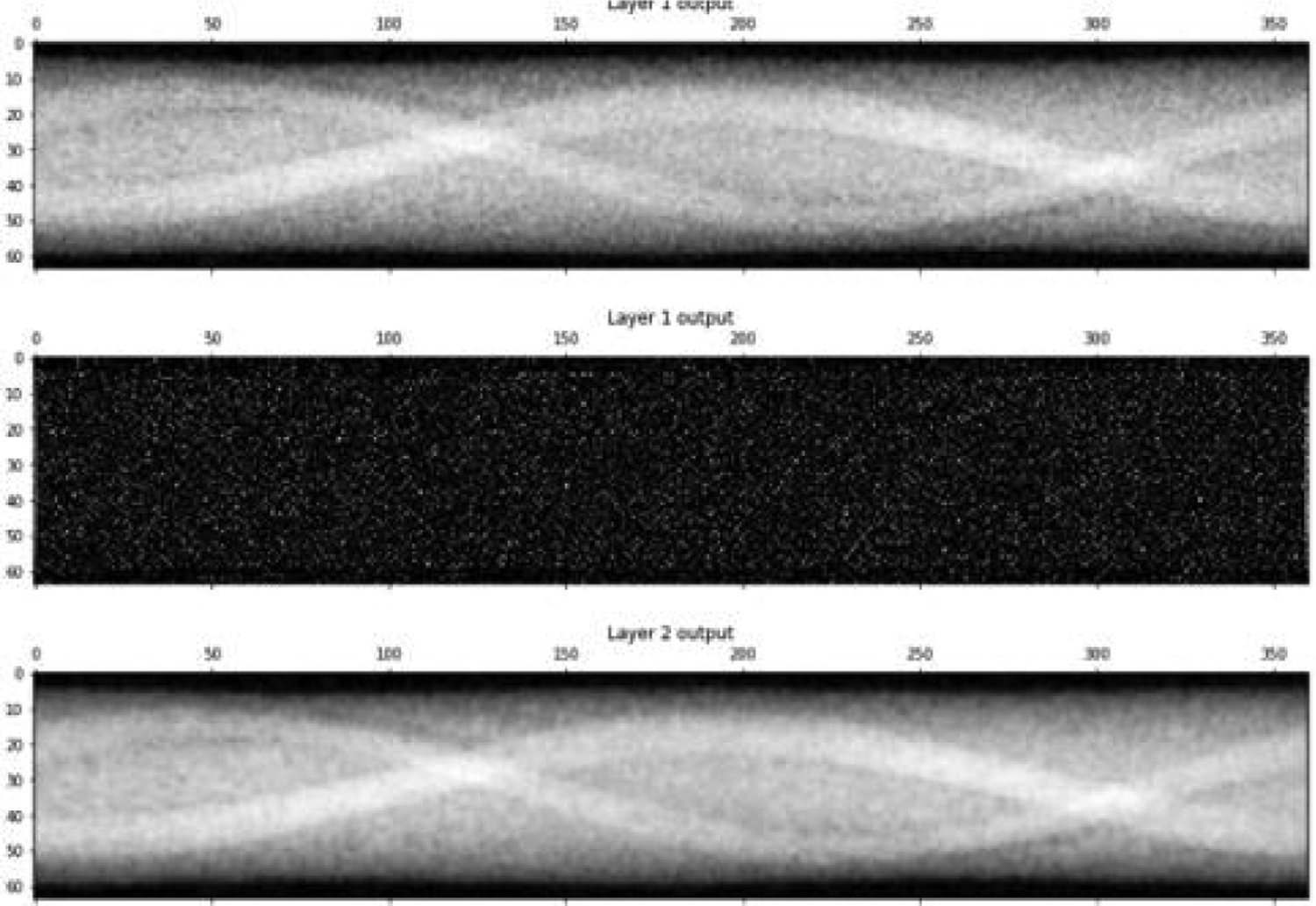
Processed sinogram outputs. Top: Low-pass channel output from the first layer. Middle: High-pass channel output from the first layer. Bottom: Final output from the second layer.

**Figure 4:**
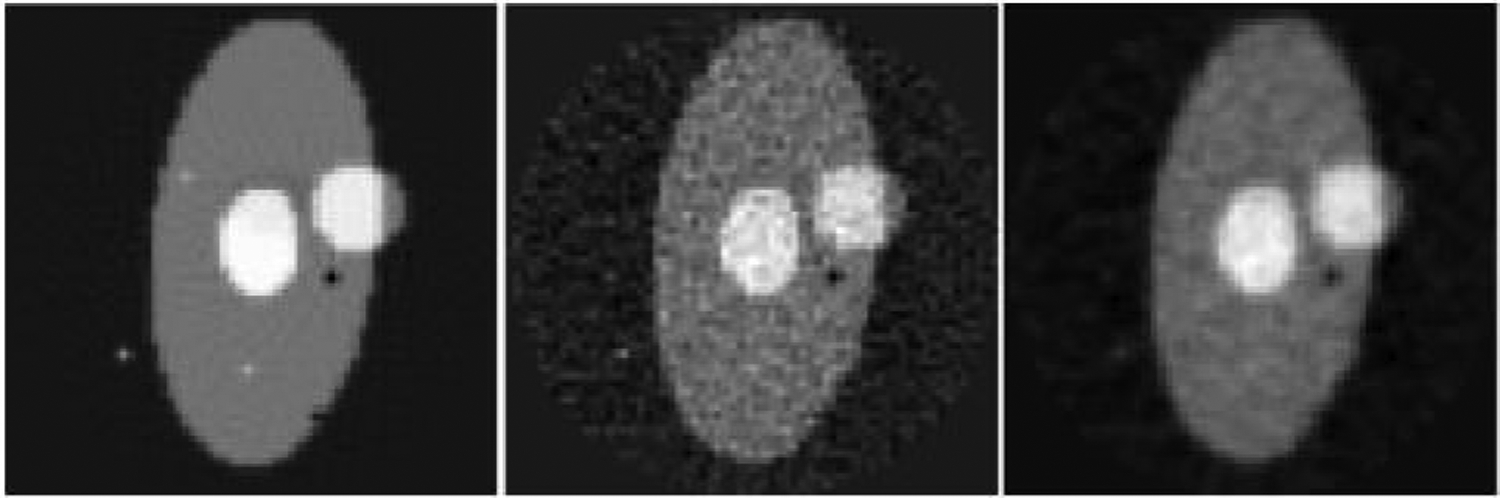
FBP reconstructed testing images. Left: Using noiseless sinogram. Middle: Using noisy sinogram. Right: Using the filtered sinogram.

A typical set of outputs of the human-designed filter are shown in Fig. 3. The low-pass channel output is similar to that from a conventional linear filter, with a minor exception that the negative values are discarded. The high-pass channel captures the salt-like noise, which will be removed by the negative sign when combining with the low-pass channel output. The high-pass channel output is new and not considered in a conventional denoising filter. The FBP reconstructions from the corresponding sinograms are shown in Fig. 4. In the test image evaluation, the MSE for the human-designed filter is 1.04 × 10^−4^, while for the machine-learned counterpart (see the first row in Table 3) is 1.42 × 10^−4^. The human-designed filter is better in our toy example (Figure 3,4).

## Conclusion

For a sinogram denoising task, this paper observes that the bias term in each neuron is unnecessary; the ReLU function is not necessary for the final output layer; using a lot of layers may not help if there is only one channel; using many channels may help if the network is shallow. We propose a human-designed 2-channel, 2-layer denoising filter for sinogram denoising (see Eq. (1)). The filter has only one design parameter u (see Fig. 2). This human-designed filter is compared with a machine-learned 2-channel, 2-layer CNN (see the first line in Table 3). The human-designed filter has a smaller MSE than the machine-learned version. Our experiments are limited, and our claims may not be valid in more general situations. However, it is innovative to adopt the state-of-the-art neural network architecture for human-designed systems.

## Figures and Tables

**Table 1:** One-channel multi-layer network performance.

Number of layers	Number of parameters	MSE
1	9	4.06 × 10^−4^
2	18	1.54 × 10^−4^
3	27	2.93 × 10^−4^
4	36	2.31 × 10^−4^

**Table 2:** One-layer network performance.

Kernel size	Number of parameters	MSE
3 × 3	9	4.06 × 10^−4^
5 × 5	25	2.63 × 10^−4^
7 × 7	49	2.93 × 10^−4^
9 × 9	81	2.31 × 10^−4^

**Table 3:** Two-layer multi-channel network performance.

Number of channels in the first layer	Number of parameters	MSE
2	18	1.42 × 10^−4^
3	54	1.39 × 10^−4^
4	72	1.09 × 10^−4^
5	90	5.40 × 10^−5^
6	108	6.85 × 10^−5^
7	126	5.73 × 10^−5^
8	144	5.27 × 10^−5^
9	162	4.14 × 10^−5^
10	180	4.20 × 10^−5^
11	198	4.20 × 10^−5^
12	216	4.41 × 10^−5^
